# Multimodal bioimaging across disciplines and scales: challenges, opportunities and breaking down barriers

**DOI:** 10.1038/s44303-024-00010-w

**Published:** 2024-03-01

**Authors:** Johanna Bischof, Georgina Fletcher, Paul Verkade, Claudia Kuntner, Julia Fernandez-Rodriguez, Linda Chaabane, Leor Ariel Rose, Andreas Walter, Michiel Vandenbosch, Marc A. M. J. van Zandvoort, Assaf Zaritsky, Antje Keppler, Maddy Parsons

**Affiliations:** 1 Euro-BioImaging ERIC Bio-Hub, European Molecular Biology Laboratory (EMBL) Heidelberg, Meyerhofstraße 1, 69117 Heidelberg, Germany; 2 BioImagingUK and Royal Microscopical Society 37/38 St Clements Oxford, Oxford, OX4, 1AJ UK; 3 Randall Centre for Cell and Molecular Biophysics, King’s College London, Guy’s Campus, London, SE1 1UL UK; 4 School of Biochemistry, University of Bristol, Bristol, BS8 1TD UK; 5 Department of Biomedical Imaging and Image-guided Therapy, Medical University of Vienna, Vienna, Austria; 6 Centre for Cellular Imaging, Sahlgrenska Academy, University of Gothenburg, SE-405 30, Gothenburg, Sweden; 7 Euro-BioImaging ERIC Med-Hub, Institute of Biostructures and Bioimaging (IBB), Italian National Research Council (CNR), Via Nizza 52, Torino, 10126 Italy; 8 Department of Software and Information Systems Engineering, Ben-Gurion University of the Negev, Beer-Sheva, 84105 Israel; 9 Center of Optical Technologies, Aalen University, Aalen, Germany; 10 Department FHML, Maastricht University, Maastricht Multimodal Molecular imaging institute, Maastricht, The Netherlands; 11 Department of Genetics and Cell Biology, Maastricht University, Institutes CARIM, GROW, and MHeNS, Maastricht, the Netherlands; 12 Institute for Molecular Cardiovascular Research (IMCAR), RWTH Aachen University Clinic, Aachen, Germany

**Keywords:** Biophysical methods, Imaging, Microscopy, Sensors and probes, Optical spectroscopy, Diseases, Chemistry, Engineering, Optics and photonics, Physics, Biophysics

## Abstract

Multimodal bioimaging is a broad term used to describe experimental workflows that employ two or more different imaging modalities. Such approaches have been in use across life science domains for several years but these remain relatively limited in scope, in part due to the complexity of undertaking these types of analysis. Expanding these workflows to encompass diverse, emerging technology holds potential to revolutionize our understanding of spatial biology. In this perspective we reflect on the instrument and workflows in current use, emerging areas to consider and our experience of the barriers to broader adoption and progress. We propose several enabling solutions across the different challenge areas, emerging opportunities for consideration and highlight some of the key community activities to help move the field forward.

## Multimodal bioimaging: context and concepts

Multimodal bioimaging development has gathered significant pace in recent years, was recently highlighted as a ‘method to watch’^1^, and is poised to transform the way in which researchers approach spatial biology challenges. Conceptually this involves the integration of multiple imaging techniques to obtain a comprehensive view of biological structures and processes. Multimodal bioimaging combines the strengths of different imaging approaches to overcome the limitations of individual techniques and provides a more holistic understanding of the sample under investigation. ‘Multimodal’ has been used as a catch-all term to describe the integration of two or more imaging modalities, within or between different domains, including (but not limited to): Microscopy Techniques (light and electron microscopy, atomic force, etc.); Molecular Imaging (fluorescence, bioluminescence, positron emission tomography (PET), etc.), Structural Imaging (X-ray, computed tomography (CT), magnetic resonance imaging (MRI), ultrasound (US), etc.); and Spectroscopy (Raman, infrared, magnetic resonance). Combining these modalities has the potential to revolutionize biological, biomedical, and clinical research by integrated visualization of complex processes in context from single molecule to whole organism.

Broadly speaking there are two categories of multimodal bioimaging: direct correlative and indirect. In *direct correlative multimodal bioimaging*, two or more imaging techniques are used simultaneously (as in hybrid hardware-fused imaging platforms) or sequentially to capture complementary information about the same biological sample and region of interest. This is valuable for understanding the relationships between different cellular or tissue structures and functions. Examples of this include correlative light electron microscopy (CLEM; data acquired sequentially on the same sample) or PET/MRI scanners (images acquired simultaneously on one multimodal instrument). *Indirect multimodal bioimaging* uses different imaging modalities to study the same biological sample type, but not necessarily at the same time or region within that sample. This approach is useful when it is challenging to synchronize modalities, or when each modality must be acquired independently due to technical (e.g., sensitivity issues or need of moving the sample to different equipment) or sample preparation constraints. Data is collected separately using each modality, and the integration between the datasets is established post-acquisition through computational or analytical techniques to enable a more holistic understanding of the sample. The level of integration needed is determined by the specific research question at hand. Examples include serial tissue sections imaged using color/fluorescence microscopy and Raman spectroscopy, or MRI and multiphoton imaging of pre-clinical models. We refer readers to several recent reviews providing detailed descriptions of technology and uses across different biological domains^2–6^.

Technological advances in the past decade have expanded capabilities in single instruments and development of workflows have improved the depth of information gained within imaging domains. Indeed, some forms of multimodal imaging (largely within specific imaging domains) have been in use for several years with impactful outcomes. However, progress in combining modalities across diverse imaging domains and disciplines (i.e.: microscopy, molecular, structural and spectroscopy) has been hampered by several confounding factors, limiting the potential to fully realize the benefits of indirect multimodal bioimaging that span multiple distinct domains. Here we provide our perspective - based on our experiences across all multimodal imaging domains - on the opportunities and challenges faced by users and developers and offer potential solutions to begin to break down these barriers for broader community adoption.

### Hardware and sample preparation challenges

The combination of imaging techniques across the multimodal domain holds significant promise to bridge the gap between structure and function, offering means to image the entire biological repertoire scaling from single molecules (lipids or proteins) to organs. Several examples already exist where different imaging disciplines have converged into single platforms or have been combined through development of appropriate tools and workflows. These have largely focused on biomedical or clinical applications thus far, but with potential for adoption into developmental biology, plant biology, and 3D culture models.

Direct correlative multimodal bioimaging across different domains generally requires colocation of instruments, particularly when using ex-vivo samples. However, such instruments are rarely located in the same facility, making workflows challenging for end users. Ideally, operating on a single multimodal instrument would circumvent these problems. The technical specifications of microscopy and molecular/structural imaging approaches make combined platforms highly challenging^7^. Some examples where these constraints have been overcome include combined optical projection tomography and light sheet microscopy to provide enhanced contrast for model organism imaging^8,9^ and coupling of quantitative fluorescence endoscopy with MRI in human and preclinical imaging to improve assessment of therapeutic response^10^. Combined optical coherence tomography (OCT) with nonlinear optical imaging and spectroscopy also enables rapid label-free imaging of structural and chemical detail in tissues^11^. For indirect multimodal bioimaging, combining in vivo and ex vivo images pose challenges through changes to organ size, structure, and morphology following sample extraction. Sensitive organs and tissues such as vessels can be carefully maintained ex-vivo under physiological conditions to mitigate this^12^. In cases where tissue fixation or dehydration are required, the use of markers that are visible across modalities is critical to ensure accurate co-registration and interpretation of features across diverse resolutions and scales.

An important issue in both direct and indirect multimodal imaging is the development of multimodal contrast agents. Such probes need to cross imaging disciplines and retain their properties to track proteins/contrast agents/cells *in vivo* and be preserved for onward analysis using complementary approaches. While such probes are being developed, they still represent a technical challenge, for example as contrast agents for MRI can interfere with downstream optical methods; microscopy-compatible contrast agents are needed to enable higher quality MR detail coupled to subcellular resolution feature retrieval. Multimodal optical/MRI contrast agents such as Gadolinium-loaded and targeted quantum dots^13,14^ are highly fluorescent and bleaching-resistant but can be toxic, limiting use of these agents to animal models. Alternatively, US and multiphoton imaging (MP) have been combined to study vessel healing in mice after injury using targeted multimodal US-MP micro-bubbles. US imaging in the living mouse revealed uptake and adhesion of the agents in vascular structures, but only the combination with *ex vivo* and *in vivo* multiphoton imaging allowed determination of the exact location of the attachment of the agents, revealing sites of damage at the molecular level^15,16^. X-ray imaging can also provide valuable structural information and when combined with bioluminescence these modalities can reveal location and function of genetically modified cells or tissues^17^.

Distinct imaging molecules/markers are not an optimal approach as each imaging agent could exhibit different *in vivo* biodistribution and pharmacokinetic properties. Single markers would help to achieve reliable bimodal probes that are comparable between imaging modalities. Recent improvements in fluorinated imaging probes for MRI has enabled dual structural imaging and tracking of immune cells *in vivo* in a mouse model of neuroinflammation, followed by detection of the same probe and chemical features at higher resolution using Raman imaging of ex-vivo spinal cord slices^18^. This allowed for real-time tracking of inflammatory cells within specific anatomical sites, coupled to single-cell level environmental chemical signatures that helps to distinguish healthy and diseased tissue. Similarly, PET/MRI imaging of glioma mouse models has been combined with optical imaging of ex-vivo optically cleared brain slices to enable co-registration of modalities and assess disease heterogeneity across scales^19^.

Multimodal approaches that bridge *in-vivo* and *ex-vivo* imaging across scales from preclinical imaging to microscopy are - despite their great potential for research and diagnostics - hampered due to the difficulties in software and hardware solutions to locate the same imaging region after transfer between platforms including correlative probes and fiducial markers. An example of such a multimodal workflow combines MRI, PET, CT, US, OCT and light microscopy to visualize vasculature at different length scales and molecular information on hypoxia and blood flow^20^. However, the workflows to integrate such datasets to retrieve impactful findings remain complex, slow and highly challenging to navigate for non-expert researchers. A summary of suggested considerations to overcome these challenges is provided in Box 1.

Box 1 Practical considerations to address multimodal workflow challenges

1. Plan experiments well in advance with experts in the relevant imaging domains, ensuring practical factors such as sample preparation, fiducial markers, probe viability, sample transfer between instruments, data correlation and analysis are all considered – along with any critical controls that might be required for each modality.
2. If more than one imaging modality will be carried out on the same sample, the order of acquisition should be evaluated to ensure samples are not comprised (e.g.: through photobleaching, reduced viability, sample destruction etc.) between instruments.
3. Discuss the desired endpoints from the multimodal images with computational experts in advance to ensure workflows and acquisition parameters will be suitable for onward analysis. Community forums such as Image.sc can help to highlight existing analysis tools for use or modification.
4. Consider location of instruments to be used; if not co-located, ensure technical staff across imaging domains discuss experimental workflows - and appropriate transit, timing and storage of samples are agreed across sites. Existing multimodal open access instruments and facilities such as those within Euro-BioImaging should be considered where feasible.
5. Define during experimental acquisition where and how data will be stored post-acquisition (nomenclature, data format, both personal and open access repositories) and ensure all relevant protocols and metadata associated with each domain are gathered.
6. For new workflows, small scale pilot experiments are advisable to enable iteration and optimization of both data acquisition and analysis and ensure that one image acquisition type does not interfere with another.

### Integration with complementary orthogonal spatial ‘omics’ approaches

‘Spatial biology’ is witnessing a significant expansion beyond the four ‘traditional’ imaging domains (introduced above) to include high-resolution spatial ‘omics’ profiling. This includes techniques such as spatial genomics/transcriptomics, imaging mass spectrometry (proteomics, metabolomics, lipidomics, metallomics etc.) and imaging mass cytometry (IMC) that provide orthogonal, highly complementary molecular detail for any sample of interest^21^. It is technically feasible to acquire directly correlated multimodal or ‘omics’ datasets on sections from tissues and more ‘classical’ biological imaging modalities on adjacent sections for co-registration. Similar challenges exist with these datasets as for other modalities, in terms of sample preparation and resolution differences. However, datasets retrieved from spatial ‘omics’ approaches can contain tens to many thousands of measurements within each pixel, making interpretation and visualization even more complex. Commercial platforms are being developed to enable easier handling of ‘omics’ spatial data with other modalities and are likely to expand significantly in coming years – ideally within the public domain - as uptake of such approaches increase.

Ultimately, combining such deep ‘omics’ phenotyping data with structural (e.g., MRI, X-ray), fluorescence (e.g., lightsheet, super-resolution) and atomic-level (e.g., volume EM) approaches will provide means to achieve a ‘Google earth’ view of any given sample, providing unprecedented insight into cell and tissue states. Creation of such holistic, rich and complex datasets for open access mining – with the ultimate goal of generating spatial tissue ‘atlases’ from different organisms and disease states – would represent extremely valuable resources for the community and open doors for new AI-based interrogation with potential impact in discovery science and therapeutic development.

### Data analysis challenges

Multi-modal analysis typically includes two main steps, co-registration, and data-fusion. Co-registration is the alignment of different modalities to a common coordinate space and enables mapping of spatial regions and/or their derived readouts from one modality to another. A variety of general-purpose registration algorithms exist that minimize the registered images discrepancy in terms of their pixel intensities, contours, features, fiducial marker localization, or point cloud alignment^22^, under a set of assumptions and parameters, ideally using software tools that unify these different approaches^23–30^. Single modality registration, for example aligning two spatially consecutive pathology slides, presents inconsistencies between the images that are being aligned as they contain information from physically different tissue sections. Further registration challenges exist due to the images used being acquired with different technologies and encoding different physical properties. These include differences in spatial resolution, field-of-view size, tissue morphological deformations due to sample preparations, moving between different imaging facilities, and imaging artifacts. These challenges require tailored solutions that reflect the specific properties of the modalities that are being interpreted and of the downstream analyses, such as selecting visually distinct structures that are shared across modalities as fiducial markers or introduction of external fiducials.

Cross-modality data fusion can be performed by spatially matching tissue regions imaged with different modalities, providing direct insight regarding the complementary information and inter-relations between the different modalities. The higher bounds (i.e., optimal) spatial scale of the matching is determined by the spatial resolution of the more coarse-resolved modality. However, in practice, the size of the spatially matched regions used for downstream analysis should be determined by the estimated registration error between the modalities. In cases where sub-sampling of different regions of the tissue using each modality has occurred, acquisition of auxiliary images (such as a brightfield image) of the full tissue along with the partial field of view can help with co-registration. Registration error can be estimated globally or locally, based on biological and physical fiducial markers that are consistent across modalities. Smaller registration errors lead to higher resolution of the cross-modality matching enabling to gain biological insight at finer spatial details. Moreover, multimodal datasets are of limited size, because the number of independent observations is limited by the costs and labor, both in terms of access to samples and the technology used to acquire them. Thus, minimizing the registration errors, both via appropriate modality selection, experimental and algorithmic design, is especially crucial for increasing the number of independent matched observations that can enable use of data-hungry machine learning analyses.

Different single cell spatial ‘omics’ technologies can be harmonized to one file-format to extract spatial statistics^31–33^. Recent studies have successfully co-registered immunofluorescence, IMC and pathology (H&E) images^34^, and IMC and imaging mass spectrometry (IMS)^35^. Unsupervised or supervised identification of image regions with a consistent biological interpretation in one modality can be used to analyze their relationships to the matched modality. Complementary information in one modality can also be used to enhance the performance of a specific task, such as improving cell type classification by using (fluorescence-based) cell segmentation^34^. Effective cross-modality data fusion requires common file-formats to enable consistent APIs across modalities^33,36^ and can take place at different stages of the information extracted from each modality, for example, raw image data, image embeddings and/or image-derived features^37,38^.

On a practical level, data acquisition from multiple experimentalists, laboratories and instruments can add to the complexity of combining such multimodal/’omics’ outputs^39^ and requires extensive quality control, harmonisation and iteration across acquisition sites. As for other multimodal imaging approaches, tools for cross-modality visualization^28^ and interpretation of the mapping between modalities^40^ are crucial for effective data exploration. In some cases where one modality contains corrupted or missing data points, these can be computationally imputed using their relationships to other modalities^41,42^. However, such imputation of missing values should be performed with great care due to potential erroneous artefacts.

Deep learning is a class of modern machine learning techniques (also known as “Artificial Intelligence”, or AI) that excel in data-driven nonlinear optimization. A key challenge in applying AI to the domain of multimodal bioimaging is the limited size of the datasets in this domain that hamper the potential of learning complex patterns in high dimensions from the (inaccessible) true distributions of these data. However, while the number of samples (e.g., patient cohort size) is limited, each sample contains a wealth of biologically relevant spatial information that enable effective application of AI, for example for single modality disease state classification, at the spatial scale of pixels^43^, cells^44^, or localized regions^45^. Pooling multiple localized regions where sample numbers are limited has already shown promise for analysis of multimodal bioimaging^35,40–42^. One domain where AI has been successfully applied to multimodal biomedical imaging datasets is radiomics. This is a quantitative approach that applies advanced mathematical analysis to extract non-visible features from medical imaging datasets and enhance traditional analysis methods. Radiomics has been further expanded through adoption of AI techniques to enable extraction of the relevant disease-specific information across large datasets. For example, to combine data from MRI, CT and/or PET images and apply different machine learning methods to extract features that are not identifiable using other means for development of predictive models^46^. Although promising, these tools are not yet in clinical use for diagnostic purposes. This is in part due to the lack of standardization in data types, lack of reproducibility across the models used and lack of agreed protocols across the field^47^.

Alongside these technical challenges, a critical factor is the continuous communication and transparency between all parties involved. In many cases, each modality is acquired and analyzed by a distinct expert, the integration of multiple modalities requires coordination between all data generators and analysts. For example, jointly defining proper acquisition of auxiliary data that will be most suitable for co-registration, characterizing the data acquisition in an optimal manner for down-stream analyses, identifying on-the-fly discrepancies in the data generated, or avoiding incorrect assumptions regarding the data. These all require coherent team communication across disciplines and a shared understanding of the ultimate goals.

### Dedicated infrastructure, community and training are critical to lower barriers

Multimodal imaging beyond the classically combined methods (CLEM) and for orthogonal modalities (as outlined above), largely requires expensive equipment and very diverse, advanced expertise in sample preparation, data acquisition and data integration. This makes it very challenging for most labs to host complex multimodal imaging pipelines and even individual institutions will struggle to have sufficient equipment and staff to provide a comprehensive set of different multimodal combinations. Undertaking these experiments therefore benefits tremendously from coordinated and quality-managed open access infrastructure, transparent processes for access and collaboration across facilities, and joint development of technologies and workflows. Euro-BioImaging ERIC (European Research Infrastructure Consortium) represents such a forum, and coordinates access to more than 190 imaging facilities across Europe. By bringing together these imaging facilities and experts across all imaging domains into one organization with a single-entry point, Euro-BioImaging facilitates and supports advances in multimodal imaging, connecting the historically siloed biological and biomedical imaging communities. This includes the establishment of mixed Nodes within Euro-BioImaging which combine expertise on biological and biomedical imaging technologies within a single entity and focus on facilitating multimodal and correlative imaging, as well as hosting regular seminars and workshops with technical specialists across domains to share knowledge and develop new approaches. Such forums are critical for sharing best practice and enabling dissemination of information to end users to overcome workflow challenges (see Box 1). However, it remains the case that most researchers and technicians are only trained in their specific imaging domain. Thus, delivery of multimodal services requires dedicated staff training and exchange of experience between experts in different imaging modalities to facilitate communication and collaboration between the different experts delivering components of multimodal workflows. Users also require advanced training and education to increase awareness of how multimodal imaging can be applied to their research and enable them to judge the strengths and limitations of multimodal imaging approaches.

This highlights the important function of coordinated research infrastructures in fostering community-driven approaches to democratizing access to - and appropriate use of - advanced imaging technology. In recent years building community initiatives and knowledge exchange forums has demonstrated to be critical for the advancement of technology, avoiding duplication of efforts, and reaching broader potential user bases. The collaborative research network COMULISglobe promotes multimodal imaging and analysis across scales from biological research to clinical diagnostics. More specifically, COMULISglobe identify, fund, and showcase novel multimodal pipelines, develop, evaluate, and share software^48^. Challenges remain, particularly in the need for continuous development of new workflows combining instruments and increasing recognition and uptake of opportunities that combined imaging approaches provide. Existing public image data repositories, such as the BioImage Archive^49^ and EMPIAR^50^, already work well for deposition of certain types of correlative multimodal image data and they are actively engaged in developments to connect data with added-value databases and repositories for other data types. The development of tools to share associated code and protocols will be critical, such as the recently developed volume EM sample preparation widget (https://www.ebi.ac.uk/empiar/spw) linking to the EMPIAR data repository. These approaches would facilitate broader uptake and re-use of datasets for novel discoveries from the normally very rich datasets produced by multimodal imaging as well as the development of new analysis approaches. Training in how to prepare data and metadata appropriately and how to share appropriate data types for onward re-use and ‘data stewardship’ to help researchers is essential to lower energy barriers and encourage routine sharing of imaging data. Funding opportunities are required for technology and analysis development as well as to allow researchers to access these technologies and receive the expert training and support required before, during and after data acquisition.

### Conclusions and future perspectives

Combinations of different imaging modalities have enhanced our understanding of complex biological systems, disease mechanisms, monitoring, and early detection. This field has encouraged cross-disciplinary collaboration between experts in imaging, biology, engineering, chemistry, computation, and medicine, leading to innovative technology solutions. However, bioimaging is entering a new era, with new technologies being developed apace, and the future challenge lies in maximizing the cross-domain potential from these discoveries. We outline our key recommendations to the community to break down barriers to broader adoption of these transformative approaches (Box 2). Improving the seamless integration of different imaging modalities, software and hardware solutions that facilitate open access data fusion and interpretation will be critical to broader adoption of these techniques. Leveraging machine learning for advanced data analytics, especially for data integration and for identifying complex correlations between modalities, will become increasingly important as the data volume increases with improved workflows and automated imaging. The expert community needs to build trust in these tools and make them accessible and understandable for all end users. The creation of more hybrid imaging systems that combine multiple modalities into a single device would be ideal to simplify data acquisition and provide real-time multimodal data. Enhancements in spatial and temporal resolutions, allowing researchers to observe even smaller biological structures and faster dynamic processes, and advancements in correlated 3D and 4D (spatiotemporal) imaging will provide more comprehensive and dynamic information about biological systems, allowing for a deeper understanding of processes in living organisms.

Box 2 Key recommendations to drive advances and adoption of multimodal bioimaging

1. Build cross-imaging domain discussion forums to explore opportunities, collaborations and engage end users early in future technology development plans
2. Develop more open access infrastructure with appropriate expertise and technology to support advanced cross-domain imaging and appropriate expert training and connect these on the local, national, and international level
3. Encourage mechanisms (workshops, seminars) to inform non-expert user communities of new multimodal bioimaging approaches and potential benefits to their research.
4. Encourage the sharing of multimodal FAIR (**F**indability, **A**ccessibility, **I**nteroperability, and **R**euse) imaging datasets and software tools in the public domain to facilitate additional discoveries from rich datasets and the development of new computational analysis approaches.
5. Foster collaborations with industry partners to facilitate the development, commercialization, and distribution of multimodal imaging systems and technologies.
6. Close community engagement with funders to highlight the importance of novel instrument development and encourage dedicated funding mechanisms to enable this and their applications.

## Data Availability

No datasets were generated or analysed during the current study.
